# A Randomized, Controlled Trial of Mirror Therapy for Upper Extremity Phantom Limb Pain in Male Amputees

**DOI:** 10.3389/fneur.2017.00267

**Published:** 2017-07-07

**Authors:** Sacha B. Finn, Briana N. Perry, Jay E. Clasing, Lisa S. Walters, Sandra L. Jarzombek, Sean Curran, Minoo Rouhanian, Mary S. Keszler, Lindsay K. Hussey-Andersen, Sharon R. Weeks, Paul F. Pasquina, Jack W. Tsao

**Affiliations:** ^1^Walter Reed National Military Medical Center, Bethesda, MD, United States; ^2^Brooke Army Medical Center, Fort Sam Houston, TX, United States; ^3^Center for Rehabilitation Sciences Research, Uniformed Services University of the Health Sciences, Bethesda, MD, United States; ^4^University of Tennessee Health Science Center, Memphis, TN, United States; ^5^Children’s Foundation Research Institute, Le Bonheur Children’s Hospital, Memphis, TN, United States; ^6^Memphis Veterans Affairs Medical Center, Memphis, TN, United States

**Keywords:** phantom limb pain, mirror therapy, upper extremity, amputee, mental visualization

## Abstract

**Objective:**

Phantom limb pain (PLP) is prevalent in patients post-amputation and is difficult to treat. We assessed the efficacy of mirror therapy in relieving PLP in unilateral, upper extremity male amputees.

**Methods:**

Fifteen participants from Walter Reed and Brooke Army Medical Centers were randomly assigned to one of two groups: mirror therapy (*n* = 9) or control (*n* = 6, covered mirror or mental visualization therapy). Participants were asked to perform 15 min of their assigned therapy daily for 5 days/week for 4 weeks. The primary outcome was pain as measured using a 100-mm Visual Analog Scale.

**Results:**

Subjects in the mirror therapy group had a significant decrease in pain scores, from a mean of 44.1 (SD = 17.0) to 27.5 (SD = 17.2) mm (*p* = 0.002). In addition, there was a significant decrease in daily time experiencing pain, from a mean of 1,022 (SD = 673) to 448 (SD = 565) minutes (*p* = 0.003). By contrast, the control group had neither diminished pain (*p* = 0.65) nor decreased overall time experiencing pain (*p* = 0.49). A pain decrement response seen by the 10th treatment session was predictive of final efficacy.

**Conclusion:**

These results confirm that mirror therapy is an effective therapy for PLP in unilateral, upper extremity male amputees, reducing both severity and duration of daily episodes.

**Registration:**

NCT0030144 ClinicalTrials.gov.

## Introduction

Shortly after amputation of a limb, up to 95% of all patients report painful or non-painful neurologic symptoms, which fall into the category of either residual limb pain (RLP), phantom sensations (PSs), or phantom limb pain (PLP) (1). PSs, or non-painful sensations perceived to be emanating from the phantom limb, typically begin soon after surgery, with one-third of patients reporting these within 24 h, three-quarters within 4 days, and 90% within 6 months (2). RLP, formerly known as “stump pain,” can persist for years post-amputation in as many as 74% of patients (3). PLP, pain perceived to be emanating from the phantom limb, typically begins within 6 months after amputation and can persist for years, with prevalence rates several years after surgery as high as 85% (4, 5).

Phantom limb pain is extremely difficult to treat as demonstrated by the numerous failed medication trials (6). Further, while there are many medications used to treat PLP, most have not been tested through rigorous controlled clinical trials, and their efficacies are instead based on positive treatment response for other neuropathic pain conditions (7–31).

The use of a virtual–reality mirror-box to treat amputee PLP was first reported by Ramachandran and Rogers-Ramachandran (32). The therapy stemmed from a theory of “learned paralysis” (32). According to this postulate, after amputation the brain still transmits efferent motor commands to the limb, yet, because the limb is missing, it fails to receive afferent sensory signals confirming that the limb successfully moved. As such, the brain perceives the limb as paralyzed, and this illusion of paralysis, in turn, causes pain. The unilateral upper extremity amputees in their small case series were asked to place the intact arm and residual limb into a box with a mirror in the middle, reflecting the intact limb and creating the illusion that the amputated limb had returned. Amputees were then asked to move their intact hand while watching the reflection in the mirror, creating the illusion that the amputated limb was moving. 60% of the amputees reported an induced illusion of phantom movement, which, for some, led to PLP reduction. Subsequent research further supports the efficacy of mirror therapy. Chan et al. conducted the first randomized, controlled trial of mirror therapy compared to covered mirror and mental visualization therapies for PLP in unilateral lower extremity amputees, reporting a 93% response rate to mirror therapy (33). To date, however, there have been no controlled, randomized trials using mirror therapy to treat upper extremity amputees with PLP. Also, it is not clear if the response rate of upper extremity PLP to mirror therapy would be more similar to that reported by Ramachandran and Rogers-Ramachandran (32) or to that seen in lower extremity amputees (33). The current study was designed to replicate the Chan et al. trial, but with upper extremity amputees with PLP and to determine if mirror therapy was also as efficacious.

## Methods

### Study Design

This was a randomized control trial to analyze the effect of mirror therapy on PLP in unilateral, upper extremity amputees. Using a computer-generated number, participants were randomly assigned to three groups, either the mirror therapy or control groups (covered mirror or mental visualization therapy). The Walter Reed Army Medical Center (WRAMC), Washington, DC, USA and Brooke Army Medical Center (BAMC), San Antonio, TX, USA Institutional Review Boards gave approval for the study, and written informed consent was obtained from all participants.

### Participants

Participants were recruited from either the Military Amputee Treatment Center at WRAMC or the Center for the Intrepid at BAMC. Subjects eligible for recruitment were active duty United States Military Service members, beneficiaries, or retirees between the ages of 18 and 70. Participants were unilateral upper extremity amputees. The study was open to both males and females, but due to the limited female population of military amputees, all participants recruited were male (34). We calculated target sample size based on the 60% response rate for mirror therapy with upper extremity amputees reported by Ramachandran and Rogers-Ramachandran (32) and using McNemar’s test of equality of paired proportions (calculated using a two-sided McNemar’s test of equality of paired proportions to give 80% power to detect a difference at a *p*-value of ≤0.05). Each participant had a minimum of three PLP episodes per week and a minimum pain score on the Visual Analog Scale (VAS) of 30 mm (out of a maximum of 100 mm) at the time of screening. Participants were also screened for effort using the Test of Memory Malingering in order to exclude those with blatant exaggeration or malingering.

Subjects were excluded on the basis of concomitant traumatic brain injury, history of vertebral disc disease or radiculopathy, uncontrolled systemic disease, significant Axis I or II diagnosis, or having participated in another PLP study within 30 days preceding intended participation in this study. During the study, subjects were allowed to take analgesic medications prescribed by their physician and continued physical and occupational therapy per standard medical care for limb amputation.

### Treatment Approach

Each participant received 15 min of the assigned therapy daily for 5 days/week for 4 weeks. Participants met with a research assistant or the study investigator at WRAMC or BAMC each session to receive the treatment and to complete pain surveys.

Volunteer subjects assigned to the mirror therapy group were asked to place their intact hand in front of a vertically placed mirror in the mid-sagittal line and to perform a series of hand movements while viewing the reflected image of the intact hand and moving the phantom in a similar manner. The movements performed were abduction/adduction of the thumb and fifth finger, flexion/extension of the thumb, flexion/extension of the fingers, pronation/supination of the hand, flexion/extension of the hand at the wrist, and flexion/extension of the elbow (for trans-humeral amputees). Subjects were asked to start with slow movements of the intact hand so that the phantom hand could keep pace with the viewed reflected image and to gradually increase the range of motion of the intact hand movements if the phantom hand had limited range of motion.

The volunteer subjects assigned to the covered mirror therapy group were given a mirror to use in the same manner as the treatment group; however, it was covered with an opaque sheet to prevent viewing of the reflection of the intact limb. They then performed the same movements with both the intact and phantom limbs. The volunteer subjects assigned to mental visualization therapy group were asked to mentally visualize the phantom limb performing the aforementioned gestures without moving their intact limb and without using a mirror. Subjects assigned to the control groups were given the option of switching to mirror therapy treatment after 4 weeks (20 treatment sessions). However, because of lack of treatment efficacy or increased pain, all subjects assigned to the control groups switched after 11 treatment sessions.

### Pain Measurements

After successful screening and consent, participants underwent a baseline assessment, which included completion of the VAS. At the beginning of each treatment session, participants were asked to again complete the VAS. Additionally, participants were asked to report the frequency (number of episodes per day) and duration of PLP (total minutes per day) at baseline and treatment sessions. Total daily time when pain was experienced by the amputee was calculated by multiplying the number of daily PLP episodes by the duration of each episode.

The VAS is a simple, efficient, minimally intrusive measure of pain, which has been widely used in clinical and research settings. It has been experimentally examined and has been found to be a valid, internally consistent, and reliable measure of pain (35). The VAS consists of a 100-mm horizontal line with two endpoints labeled “no pain” and “worst pain someone could ever experience.” Subjects were instructed to mark the line at the point corresponding to their current level of pain. The distance from the left end of the line to the subject’s mark represents a numeric index of pain severity.

### Statistical Analyses

Statistical analyses were done by Sean Curran and Minoo Rouhanian. The primary outcome variable was the VAS pain score. A repeated-measures analysis of variance was conducted to test for changes in pain over the course of treatment. Where significant changes were indicated, two-tailed, paired-samples *t*-tests were conducted to locate the time-points where the scores were significantly different than baseline. Similar statistical analyses were conducted on the secondary outcome variables. Information regarding frequency and duration of pain episodes was used to calculate total daily time experiencing pain. For all tests, an alpha level of 0.05 was used.

## Results

### Participants

**Figure 1:**
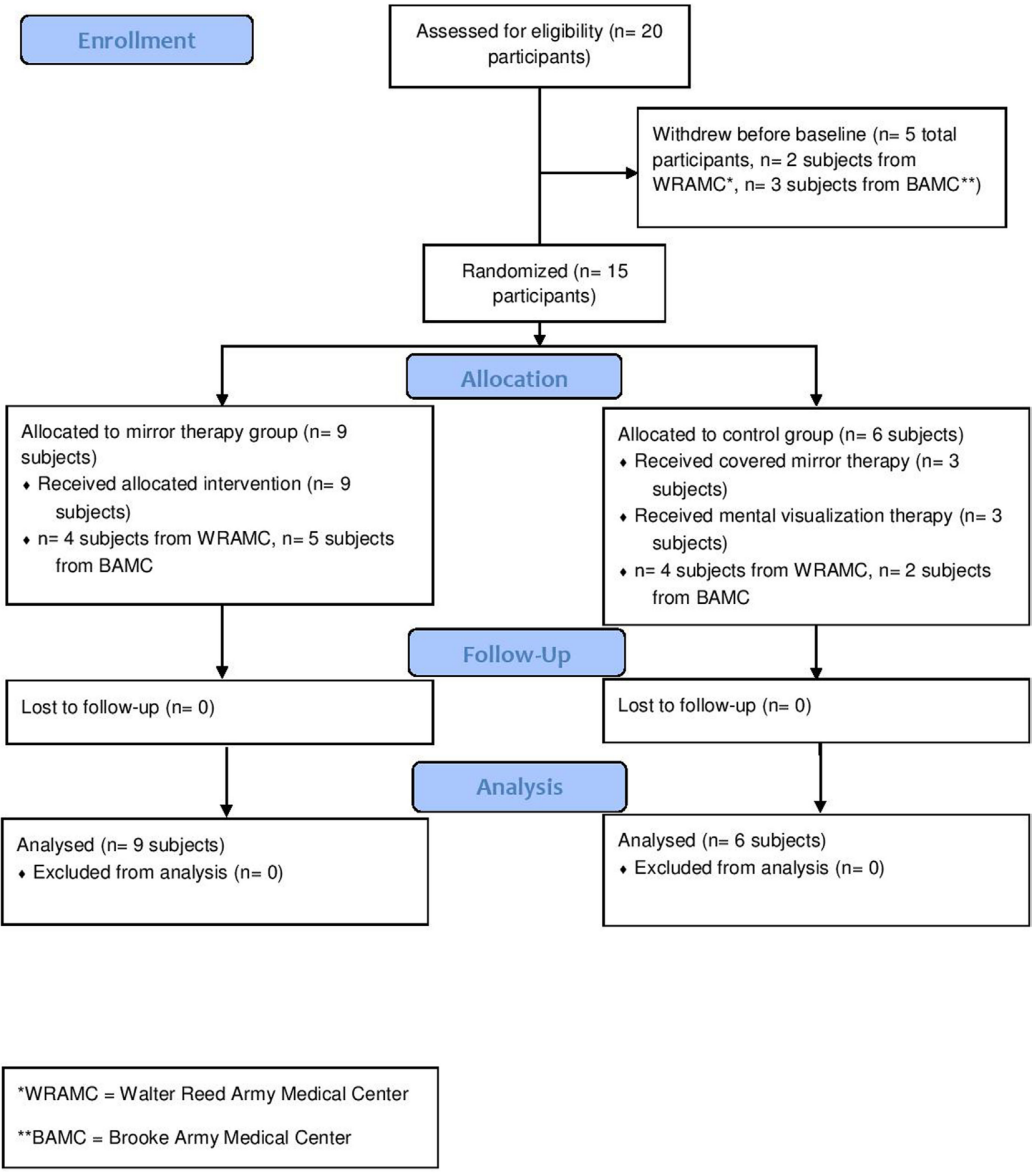
Patient flow diagram.

A total of 15 unilateral, upper extremity amputees were enrolled (Table 1). Nine amputees were randomly assigned to mirror therapy, while six were randomly assigned to the control group (three to mental visualization and three to covered mirror, combined due to small numbers) (Figure 1). All participants were using or had used gabapentin, methadone, pregabalin, and/or percocet for PLP without relief. This study was completed between August 2007 and December 2012.

**Table 1:** Participant demographic information.

	Age	Side	Site of amputation	Cause of injury	Time since injury (months)
1	25	Right	Trans-humeral	MVA	1.06
2	31	Right	Trans-radial	IED	2.29
3	22	Right	Trans-humeral	IED	0.61
4	27	Left	Wrist disarticulation	IED	0.74
5	20	Left	Trans-radial	MVA	2.00
6	68	Left	Trans-humeral	Boating accident	0.75
7	22	Left	Trans-radial	IED	4.00
8	21	Right	Trans-humeral	IED	0.55
9	19	Left	Trans-radial	IED	4.00
10	20	Right	Trans-humeral	IED	9.00
11	22	Right	Wrist disarticulation	Dynamite	11.00
12	21	Right	Trans-humeral	IED	3.58
13	22	Right	Trans-radial	IED	1.13
14	31	Right	Trans-radial	IED	3.00
15	60	Right	Trans-radial	IED	24.00

*MVA, motor vehicle accident; IED, improvised explosive device*.

### Phantom Limb Pain

**Figure 2:**
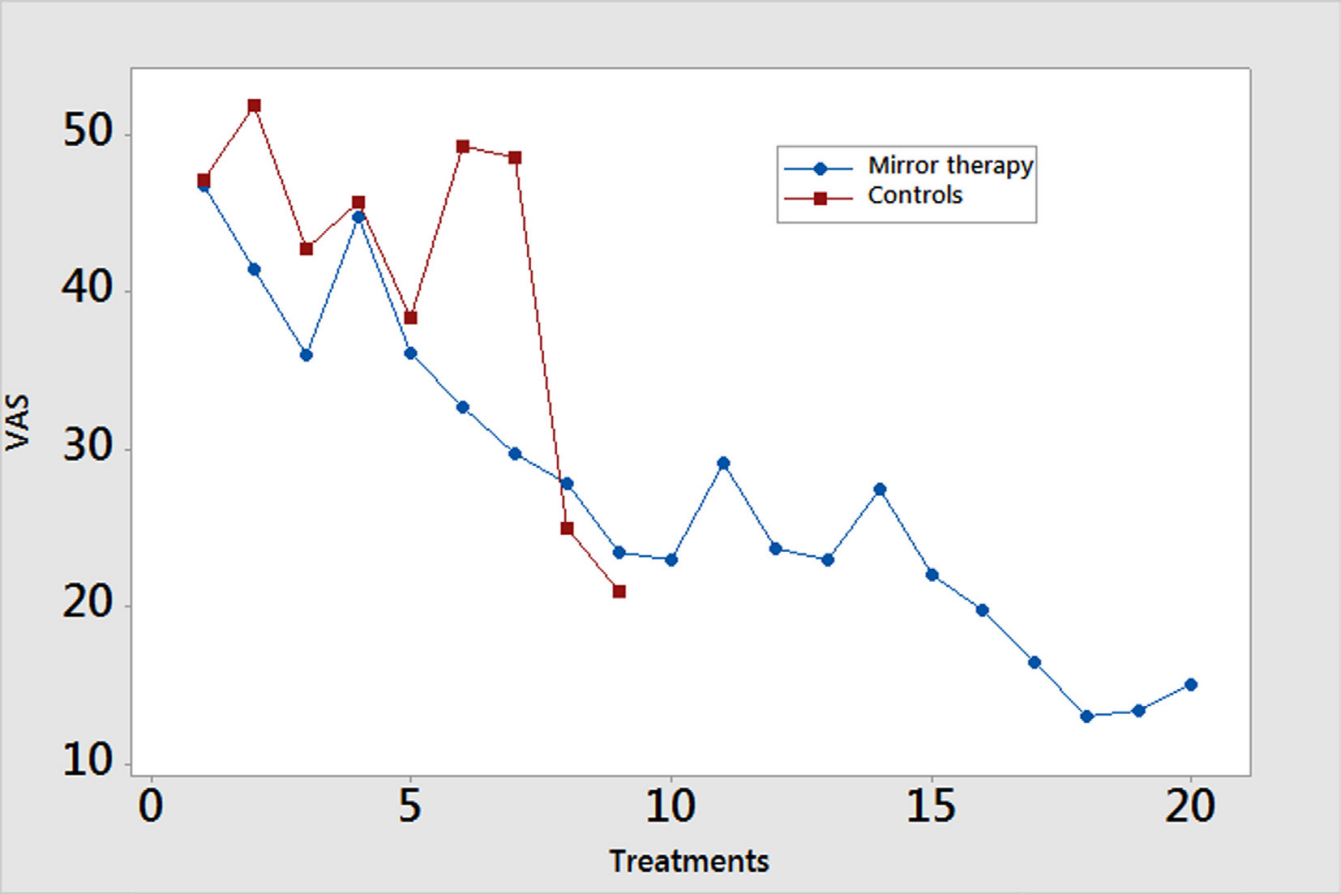
Weekly pain scores. Pain scores are reported using the Visual Analog Scale (VAS) measured on a scale of 0–100 mm. Data are presented as mean values.

In the mirror therapy group, eight amputees (89%) experienced a decrease in pain, while one subject (11%) experienced an increase in pain. The group pain score decreased from a mean of 41.4 (SD = 17.6) to 27.5 (SD = 17.2) mm on a 100-mm VAS (Figure 2, *p* = 0.001). The control group did not experience a significant reduction in pain throughout the course of treatment [mean 35.2 (SD = 25.5) to 48.5 (SD = 29.0) mm; Figure 1, *p* = 0.601], with only two subjects (20%) showing improvement. In calculating the estimated effect size of the initial and final VAS scores for those receiving mirror therapy, the Cohen’s *d* is 0.971, indicating that therapy had a large effect on pain reduction.

A study participant’s response to mirror therapy after five sessions was largely predictive of the response at 4 weeks. Six participants (66.7%) reported a directional change in their pain scores at the day 5 assessment that was consistent with their directional change after 4 weeks. Of the three remaining subjects, all reported a directional change at the day 10 assessment that agreed with that of their day 20 assessment.

### Total Daily Time Experiencing Pain

There was a significant change in total daily time spent experiencing PLP by the mirror therapy group, decreasing from a mean of 1,022 (SD = 673) to 448 (SD = 565) minutes (*p* = 0.003). Participants in the control group did not experience a significant change in daily time experiencing pain, from a mean of 743 (SD = 806) to 726 (SD = 825) minutes (*p* = 0.49). Of the seven mirror therapy subjects who initially reported constant pain, five (71%) no longer reported this at the end of treatment. In calculating the estimated effect size of the initial and final time experiencing pain per day for the therapy group, the Cohen’s *d* is 0.924, meaning that therapy had a large effect on time experiencing pain.

### Crossover Participants

**Figure 3:**
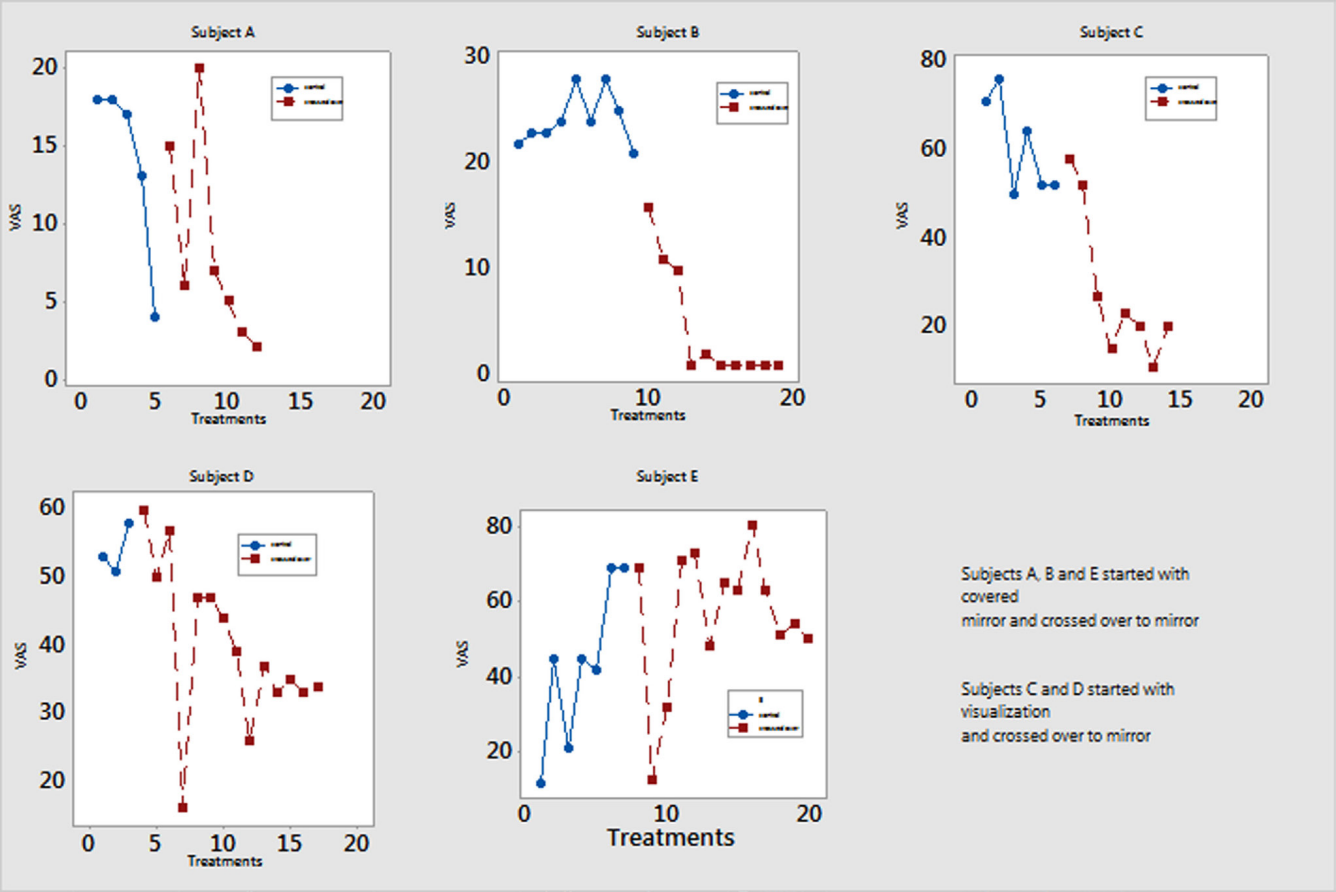
Pain scores of participants who switched from either covered mirror or mental visualization to mirror therapy. Five participants completed mirror therapy after not responding to treatment in the control group. Their Visual Analog Scale (VAS) pain scores are measured on a scale of 0–100 mm. Patient A reported decreased pain at session 5 but then had return of pain after 2 weeks and switched to minor therapy.

Five of the six patients in the control group crossed over and completed 4 weeks of mirror therapy (Figure 3). All had decreased pain severity as well as time experiencing pain.

## Discussion

This is the first randomized, controlled study of mirror therapy for treating upper extremity, male amputees with PLP. The present results support the hypothesis that the use of mirror therapy can reduce PLP in upper extremity amputees, whereas use of covered mirror and mental visualization treatments, which lack the overt visual input generated by viewing the intact limb moving in a mirror, do not significantly reduce phantom pain and may, in some instances, actually worsen pain. Interestingly, while the PLP reduction in the mirror group was significant, a subject’s response to treatment after only 5 days of therapy was largely predictive of the response at the end of therapy. PLP severity was not the only symptom found to decrease, as total daily time experiencing PLP was also significantly reduced among patients who underwent mirror therapy. Among all volunteer subjects, response by the 10th treatment session was predictive of ultimate responsiveness or lack of responsiveness to mirror therapy. As mirror therapy is not effective for all users, knowing when a response can be expected has clinical utility in defining when therapy should be changed, if necessary.

Our findings reinforce a previous case report and case series in which mirror therapy reduced PLP in upper extremity amputees (32, 36). These findings are also similar to those previously reported by Chan et al. in lower extremity amputees (33). They differ from those reported by Brodie et al. in lower extremity amputees; however, the participants in that study had only a single treatment session with mirror therapy (37). Further supporting our contention that the visual component of mirror therapy is responsible for modulating the decrease in pain are the results demonstrating that pain relief was experienced by five control subjects only after switching to mirror therapy from covered mirror or mental visualization treatments.

Visual input has been shown to influence phantom limb awareness. Hunter et al. examined this relationship in unilateral, upper extremity amputees (38). Participants were tested under the conditions of either eyes closed, eyes open, or while viewing their intact hand in a mirror, creating the illusion of a returned limb. Patients experiencing this visual illusion had the most enhanced awareness of the phantom limb, while patients tested with eyes closed were more likely to misallocate the tactile stimulation of their residual limb. In addition to visual processes, proprioceptive input and activity in the primary sensory region of the premotor cortex are believed to mediate limb perception (39). Both the success of mirror therapy in this study and the findings of both Hunter et al. (38) and Chan et al. (33) appear to support a theory that PLP is generated, in part, by a mismatch between visual and proprioceptive inputs.

The activation of mirror neurons, which fire both when an action is performed and when it is observed (40), may also contribute to therapy success by modulating somatosensory inputs and pain perception in the phantom limb. Rossi et al. demonstrated that both movement execution and observation reduce the amplitude of somatosensory-evoked potentials (41). Future research could benefit from investigating the role of mirror neurons during mirror therapy and in phantom pain relief.

There are a few limitations to this study. First, the participant population consisted only of males. The lack of females precludes generalizing the findings to all amputees suffering from PLP, as there is literature to support pain perception and pain thresholds differing between the sexes (42). Second, due to the small sample size, the study groups were could not be not divided by baseline characteristics, such as time since amputation or length of time experiencing pain. The study was designed to randomly assign participants to therapy instead of matching clinical characteristics. However, we do not believe this greatly affected our results as the initial published case series of upper extremity amputees benefiting from mirror therapy had participants who had sustained their amputations more than 10 years previously with different levels of injury (32). Other potentially confounding factors, including those unknown which might influence PLP, could not be controlled for. Finally, the findings of this study should be replicated with a larger and gender diverse population.

Importantly, these results have implications for male amputees with PLP undergoing rehabilitation, especially in areas of the world where medications are not readily available or are prohibitively expensive, since mirror therapy is a very inexpensive treatment option. Additional future considerations include a longer study timeline to better elucidate the longevity of the effectiveness of mirror therapy. Further, the effect of mirror therapy on the different subtypes of PLP should be explored.

## Ethics Statement

This study was carried out in accordance with the recommendations of both the Walter Reed and Brooke Army Medical Centers Institutional Review Boards with written informed consent from all subjects. All subjects gave written informed consent in accordance with the Declaration of Helsinki. The protocol was approved by both the Walter Reed and Brooke Army Medical Centers Institutional Review Boards.

## Author Contributions

BP, JC, LW, SJ, LH-A, MK, and SW were involved in data collection. SC and MR performed statistical analyses. SF, BP, PP, MK, and JT were involved in analyzing the results and writing the manuscript.

## Disclaimer

The opinions or assertions contained herein are the private views of the authors and are not to be construed as official or as reflecting the views of the United States Department of the Navy nor the Department of Defense.

## Conflict of Interest Statement

The authors declare that the research was conducted in the absence of any commercial or financial relationships that could be construed as a potential conflict of interest.

## References

[1] Ephraim P Wegener S MacKenzie E Dillingham T Pezzin L. Phantom pain, residual limb pain, and back pain in amputees: results of a national survey. Arch Phys Med Rehabil (2005) 86:1910–9. 10.1016/j.apmr.2005.03.031 16213230

[2] Jensen TS Krebs B Nielsen J Rasmussen P. Phantom limb, phantom pain and stump pain in amputees during the first 6 months following limb amputation. Pain (1983) 17(3):243–56. 10.1016/0304-3959(83)90097-0 6657285

[3] Ehde DM Czerniecki JM Smith DG Campbell KM Edwards WT Jensen MP Chronic phantom sensations, phantom pain, residual limb pain, and other regional pain after lower limb amputation. Arch Phys Med Rehabil (2008) 81(8):1039–44. 10.1053/apmr.2000.7583 10943752

[4] Jensen TS Krebs B Nielsen J Rasmussen P. Immediate and long-term phantom limb pain in amputees: incidence, clinical characteristics and relationship to pre-amputation limb pain. Pain (1985) 21(3):267–78. 10.1016/0304-3959(85)90090-9 3991231

[5] Davis RW. Phantom sensation, phantom pain, and stump pain. Arch Phys Med Rehabil (1993) 74:79–91. 8380543

[6] Sherman RA Sherman CJ Parker L. Chronic phantom and stump pain among American veterans: results of a survey. Pain (1984) 18:83–95. 10.1016/0304-3959(84)90128-3 6709380

[7] Knotkova H Cruciani RA Tronnier VM Rasche D. Current and future options for the management of phantom-limb pain. J Pain Res (2012) 5:39–49. 10.2147/JPR.S16733 22457600 PMC3308715

[8] Alviar MJ Hale T Dungca M Pharmacologic interventions for treating phantom limb pain. Cochrane Database Syst Rev (2011) 12:CD006380 10.1002/14651858.CD006380.pub2 22161403

[9] O’Connor AB Dworkin RH. Treatment of neuropathic pain: an overview of recent guidelines. Am J Med (2009) 122:S22–32. 10.1016/j.amjmed.2009.04.007 19801049

[10] Byrant B Knights K Salerno E Pharmacology for Health Professionals. Amsterdam, Holland: Elsevier (2007). 270 p.

[11] Attal N Cruccu G Baron R Haanpää M Hansson P Jensen TS EFNS guidelines on the pharmacological treatment of neuropathic pain: 2010 revision. Eur J Neurol (2010) 17(9):1113–e88. 10.1111/j.1468-1331.2010.02999.x 20402746

[12] Robinson LR Czerniecki JM Ehde DM Edwards WT Judish DA Goldberg ML Trial of amitriptyline for relief of pain in amputees: results of a randomized controlled study. Arch Phys Med Rehabil (2004) 85(1):1–6. 10.1016/S0003-9993(03)00476-3 14970960

[13] Sato K Higuchi H Hishikawa Y Management of phantom limb pain and sensation with milnacipran. J Neuropsychiatry Clin Neurosci (2008) 20(3):368 10.1176/jnp.2008.20.3.368 18806244

[14] Nikolajsen L Finnerup NB Kramp S Vimtrup AS Keller J Jensen TS. A randomized study of the effects of gabapentin on postamputation pain. Anesthesiology (2006) 105(5):1008–15. 10.1097/00000542-200611000-00023 17065896

[15] Spiegel DR Lappinen E Gottlieb M. A presumed case of phantom limb pain treated successfully with duloxetine and pregabalin. Gen Hosp Psychiatry (2010) 32(2):228.e5–7. 10.1016/j.genhosppsych.2009.05.012 20303003

[16] Hackworth RJ Tokarz KA Fowler IM Wallace SC Stedje-Larsen ET. Profound pain reduction after induction of memantine treatment in two patients with severe phantom limb pain. Anesth Analg (2008) 107(4):1377–9. 10.1213/ane.0b013e31817f90f1 18806054

[17] Schley M Topfner S Wiech K Schaller HE Konrad CJ Schmelz M Continuous brachial plexus blockade in combination with the NMDA receptor antagonist memantine prevents phantom pain in acute traumatic upper limb amputees. Eur J Pain (2007) 11(3):299–308. 10.1016/j.ejpain.2006.03.003 16716615

[18] Zuniga RE Schlicht CR Abram SE Intrathecal baclofen is analgesic in patients with chronic pain. Anesthesiology (2009) 92(3):876–80. 10.1097/00000542-200003000-00037 10719971

[19] Omote K Ohmori H Kawamata M Matsumoto M Namiki A Intrathecal buprenorphine in the treatment of phantom limb pain. Anesth Analg (1995) 80(5):1030–2. 10.1213/00000539-199505000-00030 7726400

[20] Bergmans L Snijdelaar DG Katz J Crul BJ. Methadone for phantom limb pain. Clin J Pain (2002) 18(3):203–5. 10.1097/00002508-200205000-00012 12048424

[21] Cruciani RA Strada EA Knotkova H Neuropathic pain. In: Bruera ED Portenoy RK, editors. Cancer Pain – Assessment and Management. New York, NY: Cambridge University Press (2010). p. 478–505.

[22] Wilder-Smith CH Hill LT Laurent S. Postamputation pain and sensory changes in treatment-naive patients: characteristics and responses to treatment with tramadol, amitriptyline, and placebo. Anesthesiology (2005) 103(3):619–28. 10.1097/00000542-200509000-00027 16129989

[23] Kuiken TA Schechtman L Harden RN. Phantom limb pain treatment with mirtazapine: a case series. Pain Pract (2005) 5(4):356–60. 10.1111/j.1533-2500.2005.00038.x 17177770

[24] Karanikolas M Aretha D Tsolakis I Monantera G Kiekkas P Papadoulas S Optimized perioperative analgesia reduces chronic phantom limb pain intensity, prevalence, and frequency: a prospective, randomized, clinical trial. Anesthesiology (2011) 114(5):1144–54. 10.1097/ALN.0b013e31820fc7d2 21368651

[25] Rasmussen S Kehlet H Management of nerves during leg amputation – a neglected area in our understanding of the pathogenesis of phantom limb pain. Acta Anaesthesiol Scand (2007) 51(8):1115–6. 10.1111/j.1399-6576.2007.01389.x 17697308

[26] Hayes C Armstrong-Brown A Burstal R. Perioperative intravenous ketamine infusion for the prevention of persistent post-amputation pain: a randomized, controlled trial. Anaesth Intensive Care (2004) 32(3):330–8. 15264726 10.1177/0310057X0403200305

[27] Wilson JA Nimmo AF Fleetwood-Walker SM Colvin LA A randomized double blind trial of the effect of preemptive epidural ketamine on persistent pain after lower limb amputation. Pain (2008) 135(1–2):108–18. 10.1016/j.pain.2007.05.011 17583431

[28] Fromm GH Terrence CF Chattha AS Baclofen in the treatment of trigeminal neuralgia: double-blind study and long-term follow up. Ann Neurol (1984) 15(3):240–4. 10.1002/ana.410150306 6372646

[29] Pandey CK Singh N Singh PK Gabapentin for refractory idiopathic trigeminal neuralgia. J Indian Med Assoc (2008) 106(2):124–5. 18705259

[30] Ehde DM Jensen MP Engel J Turner JA Hoffman AJ Cardenas DD. Chronic pain secondary to disability: a review. Clin J Pain (2003) 19(1):3–17. 10.1097/00002508-200301000-00002 12514452

[31] Perez MA Cohen LG Principles and mechanisms of transcranial direct current stimulation. In: Knotkova H Cruciani RA Merrrick J, editors. Brain Stimulation in the Treatment of Pain. New York, NY: Nova Science Publishers Inc. (2010). p. 113–29.

[32] Ramachandran VS Rogers-Ramachandran D. Synaesthesia in phantom limbs induced with mirrors. Proc Biol Sci (1996) 263:377–86. 10.1098/rspb.1996.0058 8637922

[33] Chan B Witt R Charrow A Magee A Howard R Pasquina PF Mirror therapy for phantom limb pain. N Engl J Med (2007) 357(21):2206–7. 10.1056/NEJMc071927 18032777

[34] Women in Military Service for America Memorial Foundation, Inc. Statistics on Women in the Military. Data reported by Department of Veteran Affairs, as of September 30. Washington, DC (2011).

[35] Price DD Bush FM Long S Harkins SW. A comparison of pain measurement characteristics of mechanical visual analogue and simple numerical rating scales. Pain (1994) 56:217–26. 10.1016/0304-3959(94)90097-3 8008411

[36] Sumitani M Miyauchi S McCabe CS Shibata M Maeda L Saitoh Y Mirror visual feedback alleviates deafferentation pain, depending on qualitative aspects of the pain: a preliminary report. Rheumatology (Oxford) (2008) 47:1038–43. 10.1093/rheumatology/ken170 18463143

[37] Brodie EE Whyte A Niven CA Analgesia through the looking glass? A randomized controlled trial investigating the effect of viewing a ‘virtual’ limb upon phantom limb pain, sensation and movement. Eur J Pain (2007) 1(4):428–36. 10.1016/j.ejpain.2006.06.002 16857400

[38] Hunter JP Katz J Davis KD. The effect of tactile and visual sensory inputs on phantom limb awareness. Brain (2003) 126:579–89. 10.1093/brain/awg054 12566279

[39] Christensen MS Lundbye-Jense J Geertsen SS Peterson TH Paulson OB Nielsen JB. Premotor cortex modulates somatosensory cortex during voluntary movements without proprioceptive feedback. Nat Neurosci (2007) 10:417–9. 10.1038/nn1873 17369825

[40] Cattaneo L Rizzolatti G. The mirror neuron system. Arch Neurol (2009) 66(5):557–60. 10.1001/archneurol.2009.41 19433654

[41] Rossi S Tecchio F Pasqualetti P Ulivelli M Pizzella V Romani GL Somatosensory processing during movement observation in humans. Clin Neurophysiol (2002) 113:16–24. 10.1016/S1388-2457(01)00725-8 11801420

[42] Wisenfeld-Hallin Z. Sex differences in pain perception. Gend Med (2005) 2(3):137–45. 10.1016/S1550-8579(05)80042-7 16290886

